# Islet transplantation modulates macrophage to induce immune tolerance and angiogenesis of islet tissue in type I diabetes mice model

**DOI:** 10.18632/aging.104085

**Published:** 2020-11-20

**Authors:** Yang Li, Xiaoming Ding, Xiaohui Tian, Jin Zheng, Chenguang Ding, Xiao Li, Xiaojun Hu, Yuxi Qiao, Ying Wang, Wujun Xue

**Affiliations:** 1 Department of Renal Transplantation, The First Affiliated Hospital of Xi’an Jiaotong University, Xi’an 710061, China

**Keywords:** islet transplantation, T1D, immune tolerance, macrophages, neovascularization

## Abstract

Objective To investigate the dual mechanism of islet transplantation in T1D by regulating the immune tolerance of macrophages and inducing the neovascularization.

Methods NC group, T1D model group and T1D model + islet group were constructed. Then, the abdominal aorta blood of abdominal aorta and islet tissue were collected. ELISA was performed to detect the level of IL-1Rα, IL-1α, IL-1β, CXCL2, MCP1, TNF-α and IL-10. Flow cytometry was used to measure the content of M1 and M2 macrophages. HE staining indicated the pathological characteristics of islet. IHC and WB were applied to determine the protein levels of IGF1R, FGFR2 or VEGFA as well as IGF1R, GRB2, EGFR, PTPN1, JAK2, STAT3, Caspase-1, Bcl2 respectively.

Results Islet transplantation in T1D stimulated the expression of IL-1Rα, IL-1α, IL-1β, CXCL2, MCP1, TNF-α and IL-10 in abdominal aorta blood, changed the content of MHCII^+^CD206^-^M1 and MHCII^+^CD206^+^M2 macrophages, reduced the pathological features and the infiltration of immunocytes, promoted the expression of IGF1R, FGFR2 and VEGFA, eliminated cell apoptosis and induced the neovascularization in islet grafts.

Conclusions Islet transplantation is an effective strategy for the treatment of T1D. It can increase the content of M2 macrophages whose immune tolerance can elevate the survival of islet grafts, reduce the inflammatory responses mediated by macrophages, promote the neovascularization and eliminate the cell apoptosis of islet grafts.

## INTRODUCTION

Type 1 diabetes mellitus (T1D) is an autoimmune disease. Because of the gradual destruction of islet β cells in the body, insulin secretion is absolutely insufficient [1]. The disease has a high incidence in children and adolescents mostly aged 10 to 15 years old [2]. The main clinical manifestations are polydipsia and polyuria, which are often accompanied by weight loss [3]. Investigations have shown that the etiology of T1D is complicated, and the apoptosis of islet β cells is induced by many kinds of factors, including genetic background, environment, and economic condition [4–6].

Islet transplantation is an effective treatment for T1D [7]. More and more studies have pointed out that the stimulating immunoregulatory cells such as macrophages to induce immune tolerance is an ideal adjuvant therapy for islet transplantation [8, 9]. The exact mechanisms of selectively activated macrophages or the different content of macrophage subtypes still remain largely unknown in T1D with islet transplantation, which plays a protective role in various inflammatory and immune responses.

As an important cellular component of innate immunity, macrophages are characterized by high plasticity and abnormal differentiation in response to various stimuli. In view of this, macrophages in physiological and pathological conditions show great heterogeneity. In general, macrophages can be polarized into either proinflammatory macrophages (M1) or anti-inflammatory macrophages (M2), depending on their environment. M1 macrophages have the characteristics of promoting inflammation while M2 macrophages have the characteristics of inhibiting inflammation in non-neoplastic diseases. The relative functions of the two subtypes are almost completely opposite [10]. Recent studies have shown that certain stimulated macrophage polarization may be promising therapeutic tools for treating related diseases [11]. Selective activation of M2 macrophages has a protective role in a variety of inflammations and immune responses [12]. These M2 macrophages mainly mediate Th2 by secreting various regulatory cytokines such as IL-1Rα, IL-1α, IL-1β, CXCL2, MCP1, TNF-α and IL-10 et al, and play an immune regulatory and anti-inflammatory role [13], however, the potential role of macrophage in T1D islet transplantation still needs further investigation.

As we all know, coronary heart disease, abdominal aorta and other cardiovascular diseases are very common complications of T1D, and chronic vasculitis is the fundamental basis of diabetic vasculopathy [14, 15]. Islet transplantation can repair langerhans islet injury by inducing angiogenesis, inhibiting apoptosis and promoting the islet cell regeneration [16]. However, the underlying mechanisms, risk factors and therapy strategies associated with M2 macrophages tolerance in T1D neovascularization after islet transplantation remain largely unknown and further *in vivo* and *in vitro* investigations have been paid much more attention. It was proven that the interaction between IGF1R, PTPN1, EGFR, GRB2, JAK2, STAT3, FGFR2 and VEGFA could regulate the apoptosis and proliferation of islet cells and could induce the formation of islet matrix [17]. Mounting previous reports have demonstrated that these proteins play an important role in the development of T1D after islet transplantation [18], while their contribution to the neovascularization in islet graft has never been fully understood in recent years.

Therefore, this study was undertaken to investigate and explore the dual mechanism of islet transplantation in T1D which regulates the survival of islet grafts by immune tolerance of macrophages and induces the neovascularization in islet grafts.

## RESULTS

### Islet transplantation in T1D stimulated the expression of IL-1Rα, IL-1α, IL-1β, CXCL2, MCP1, TNF-α and IL-10 in the abdominal aorta blood

**Figure 1:**
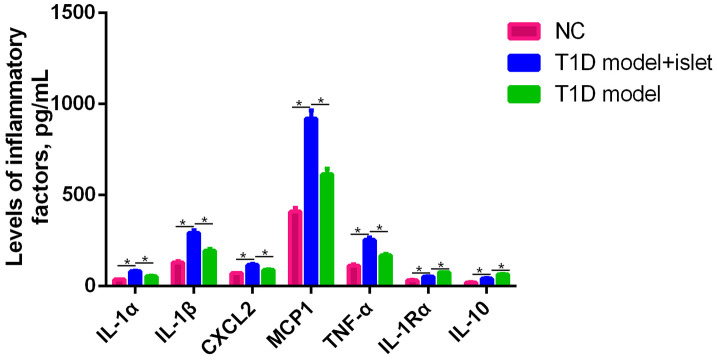
**The expression of IL-1Rα, IL-1α, IL-1β, CXCL2, MCP1, TNF-α and IL-10 in the abdominal aorta blood was detected by ELISA.** ELISA assay was performed to measure the expression level of IL-1α, IL-1β, CXCL2, MCP1, TNF-α, IL-1R and IL-10 in the abdominal aorta blood of NC, T1D model with islet transplantation and T1D model. *P<0.05.

Firstly, we screened several proinflammatory factors including IL-1Rα, IL-1α, IL-1β, CXCL2, MCP1, TNF-α and IL-10 from the abdominal aorta blood of NC, T1D model and T1D model with islet transplantation. We found that the expression levels of IL-1α, IL-1β, CXCL2, MCP1, TNF-α and IL-10 in T1D model were significantly higher than those of NC group and the islet transplantation group. While, the levels of IL-1Rα and IL-10 in the islet transplantation group were slightly increased compared to the NC and T1D model groups (Figure 1). Besides, after islet transplantation, the mean blood glucose levels of the mice decreased from 271±16.3 mg/dl to 158±10.6 mg/dl in the islet transplantation group. These results indicated that islet transplantation in T1D could induce the secretion of various proinflammatory factors as well as decrease the blood glucose.

### Islet transplantation changed the content of MHCII^+^CD206^-^M1 and MHCII^+^CD206^+^M2 macrophages in islet grafts

**Figure 2:**
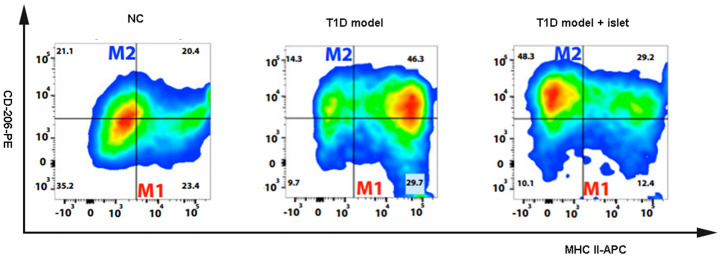
**The content of MCHII^+^CD206^-^M1 and MCHII^+^CD206^+^M2 macrophages were detected by flow cytometry.** The flow cytometry was applied to detect the macrophage subtypes including MCHII^+^CD206^-^M1 and MCHII^+^CD206^+^M2 macrophages in the different groups of NOD mice.

In the various pathological conditions, macrophages participated in inflammation and played proinflammatory or anti-inflammatory roles. Classically, macrophages were subdivided into MHCII^+^CD206-M1 and MHCII^+^CD206^+^M2 macrophages according to the local environment. In this study, we analyzed the content of macrophages in the abdominal aorta blood following islet transplantation in T1D model and found that of the M1 macrophages were significantly increased in the T1D model with islet transplantation compared to that of NC. However, the proportion of M2 macrophages in the T1D model with islet transplantation was higher than that in the NC and T1D model. In the NC group, there was no significant difference in the ratio of M1 to M2 macrophages (Figure 2). These results showed us that in the T1D model, the content of macrophage subtypes might be associated with islet transplantation, suggesting that the proinflammatory phenomenon mentioned in Figure 1 maybe due to alterations in macrophage subtypes.

### Islet transplantation reduced the pathological features in islet grafts and decreased the infiltration of immunocytes

**Figure 3:**
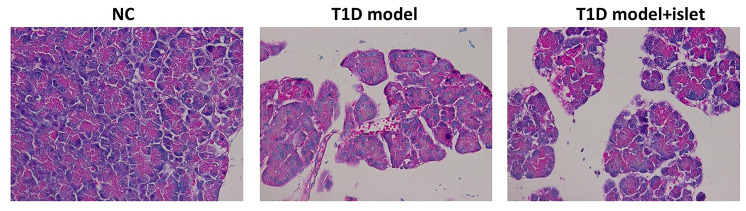
**The pathological features of islet in each group were analyzed by HE staining.** HE staining indicated the pathological features and the infiltration of immunocytes in each group.

IHC results further proved that islet transplantation in the T1D model obviously lenified the pathological characteristics and immunocytes infiltration in the islet grafts compared with the T1D model while no change was observed in the NC group (Figure 3). This phenomenon told us that islet transplantation should retard the pathogenesis of T1D and decrease the inflammation in islet grafts.

### Islet transplantation promoted the expression of IGF1R, FGFR2 and VEGFA in islet grafts

**Figure 4:**
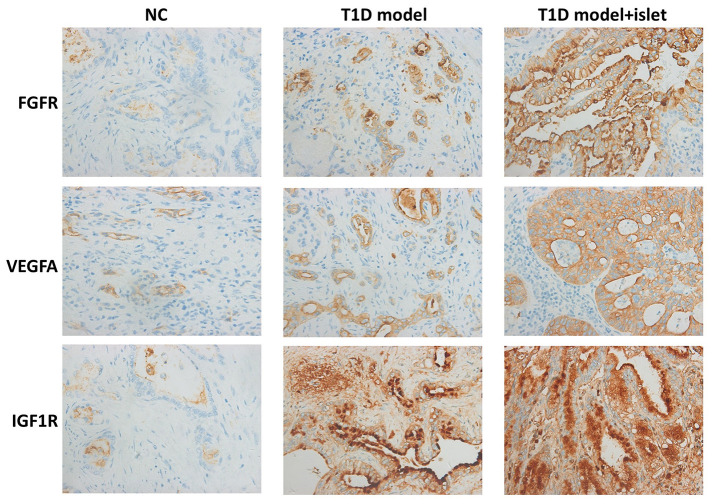
**Immunohistochemical analysis of IGF1R, FGFR2 and VEGFA in islet grafts.** IHC demonstrated the protein level of IGF1R, FGFR2 and VEGFA in the cryosection of islet grafts.

To investigate the effects of islet transplantation on the neovascularization in islet grafts, we examined the expression of IGF1R, FGFR2 and VEGFA in islet grafts by IHC. There was a great amount of FGFR as well as VEGFA in the extracellular matrix while IGF1R mainly located in the cytoplasm. And their expression was much higher in T1D model with islet transplantation than that of the T1D model and NC group (Figure 4). These results demonstrated that islet transplantation in T1D model could promote neovascularization through increasing the protein expression of IGF1R, FGFR2 and VEGFA.

### Islet transplantation eliminated cell apoptosis and induced the neovascularization in islet grafts

**Figure 5:**
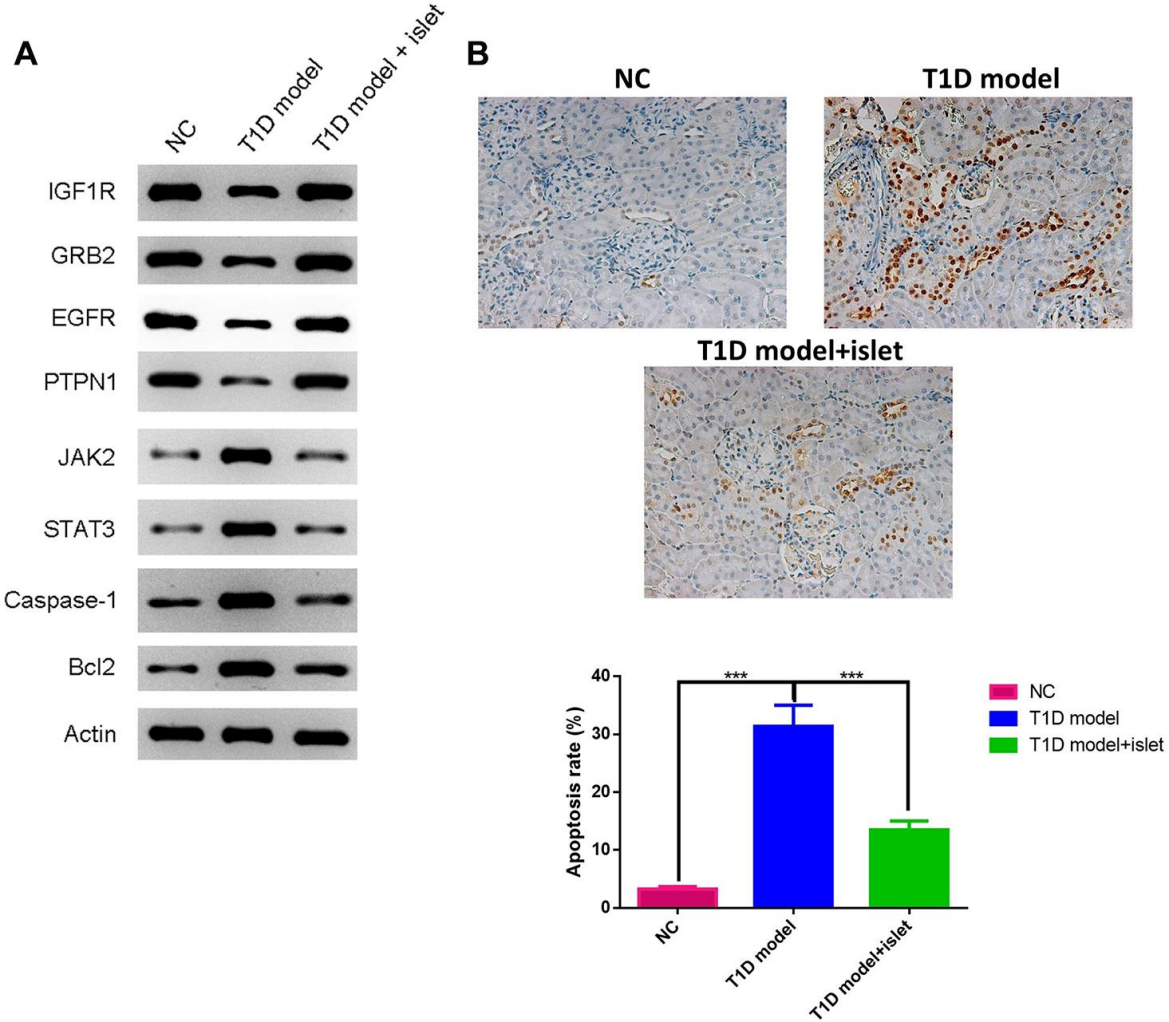
**The expressions of IGF1R, GRB2, EGFR, PTPN1, JAK2, STAT3, Caspase-1 and Bcl2 in islet grafts was analyzed by WB and cell apoptosis by TUNEL.** (**A**) The protein expression of IGF1R, GRB2, EGFR, PTPN1, JAK2, STAT3, Caspase-1 and Bcl2 in islet grafts was detected by WB. (**B**) TUNEL staining for different groups.

To further unveil the effects of islet transplantation in islet neovascularization and cell apoptosis, we performed WB to quantitatively analyzed the protein level of IGF1R, GRB2, EGFR, PTPN1, JAK2, STAT3, Caspase 1 and Bcl2 in islet grafts. We found that IGF1R, GRB2, EGFR and PTPN1 were significantly increased in T1D model with islet transplantation while JAK2, STAT3, caspase-1 and Bcl2 were remarkably decreased when compared with T1D model (Figure 5). TUNEL staining also showed the apoptosis rate was remarkably higher in T1D model group, which was attenuated by islet transplantation. These results proved that islet transplantation not only promoted neovascularization but also eliminated cell apoptosis in islet grafts.

## DISCUSSION

This original article of T1D in NOD mice shows that the 10-week-old female NOD mice with T1D after islet transplantation presented with a decreased secretion of IL-1α, IL-1β, CXCL2, MCP1 and TNF-α while an increased secretion of IL-1Rα and IL-10 when compared to the T1D model. ‘Moreover, there was a great increase in M2 macrophages in the abdominal aorta blood of T1D mice with islet transplantation led to the elimination of immunocytes infiltration and pathological features. In islet grafts, the neovascularization was enhanced significantly while cell apoptosis was reduced a lot due to the increased expression of FGFR, VEGFA, GRB2, PTPN1 and IGF1R as well as the decreased expression of JAK2, STAT3, caspase-1 and Bcl2. Thus, the achievement of this current results was prone to be crucial for exploring the dual mechanisms of islet transplantation and T1D clinical treatment.

Mounting observational studies have identified type 1 diabetes mellitus (T1D) as an insulin-dependent autoimmune disease [19]. Genetic background and immune tolerance defects are the main causes of the disease [20, 21]. A safe and effective immune tolerance induction program has always been the goal pursued by transplant physicians. The use of islet cells or tissues with immunoregulatory functions to induce immune tolerance to the grafts can reduce or eliminate immunosuppressants completely, thereby avoiding the long-term use of immunosuppressants [22, 23].

As an important component of human innate immune system, macrophages have complex heterogeneity and functional diversity due to tissue distribution, differentiation and diversity of external activators [24]. In 1992, Siamon Gordon’s team first proposed the concept of “alternative activated macrophages” (AAMs or M2), and found that the Th2 cytokine IL-4 induced macrophages to produce a phenotype different from that of INF-γ activated phenotype and it was characterized by glycosylated ligands that increased endocytosis and enhanced MHC class II antigen expression, and reduced secretion of proinflammatory cytokines. Unlike classically activated macrophages (CAMs or M1), M2 macrophages secreted high levels of IL-10 and other chemokines including IL-1Rα, which reduced their ability to present antigens and kill intracellular pathogens [25, 26]. M2 macrophages could induce host immune non-response and prevent M1 inflammatory macrophages from exerting a killing effect. It was independent of the protective mechanism of lymphocytes and preferentially aggregates in inflammatory lesions in the body. The accumulation of exogenous macrophages in the injured area and down-regulation of inflammatory cytokines played a protective role. In addition, M2 macrophages had abundant sources (ascites, spleen, bone marrow, peripheral blood, etc.), and the *in vitro* isolation and induction technology was mature, suggesting that M2 macrophages could also be one of the ideal cell therapy methods for immune regulation [27]. However, the changes of macrophage subtypes after islet transplantation in T1D mice has never been reported until recent years. Based on these previous reports, this study further unveiled a significant increase in M2 macrophages in the T1D mice after islet transplantation.

On the other hand, M2 macrophages played an important role in tissue repair, graft protection, and prolonged graft survival. Some studies have found that local sustained-release of dexamethasone in the grafts can promote islet grafts survival in diabetic mice by inducing differentiation of M2 macrophages in the microenvironment of the grafts and secretion of anti-inflammatory factors. In our study, we utilized ELISA to measure the secretion of various proinflammatory factors and the results demonstrated that only IL-10 and IL-1Rα were increased in the T1D model after islet transplantation and IL-1α, IL-1β, CXCL2, MCP1 and TNF-α decreased significantly, indicating that islet transplantation in T1D mice exerted anti-inflammatory roles due to the mysterious reasons. The pathological sections of grafts found that M1 macrophages was mostly present in the grafts with acute rejection, while a large number of M2 macrophages were found in the non-rejected grafts, suggesting that M2 macrophages had an effect on the cornea [28]. Similarly, our results found that in T1D mice after islet transplantation, the pathological features and immunocytes infiltration has been eliminated to a large extent, proving that islet transplantation could induce the maturation of M2 macrophages and reduce the pathogenesis of T1D *in vivo*. And we proposed that islet transplantation in T1D mice have induced the generation of M2 macrophages *in vivo*, thus protecting the grafts and confirming the ability of M2 macrophages to inhibit the inflammatory response of the grafts.

The Phipps’ team transplanted bone marrow-derived M2 macrophages induced *in vitro* with autologous fat grafts and transplanted them back to mice. Their results showed that M2 macrophages could improve revascularization or neovascularization of the grafts, thereby improving the survival conditions of fat grafts, M2 macrophages were thought to have a repair function in the graft response [29]. To date, however, there have been no reports of islet transplantation *in vivo* using M2 macrophages to observe their effects on neovascularization in T1D mice. However, our study found a fascinating finding, and firstly reported that islet transplantation could promote neovascularization and eliminate islet cell apoptosis in T1D mice. This phenomenon could pave the way for resolving the underlying molecular mechanisms of M2 macrophages on neovascularization in T1D mice after islet transplantation.

## CONCLUSIONS

As mentioned above, our study delivered an important discovery and firstly showed the dual mechanism of islet transplantation through M2 macrophages in T1D. In T1D mice, islet transplantation not only reduced the proinflammatory response and pathological features, but also stimulated the neovascularization and decreased cell apoptosis in islet grafts. This study could provide very important fundamental thesis for clinical application of T1D therapy.

## MATERIALS AND METHODS

### T1D mice model and grouping

Briefly, 12 female 4-week-old NOD mice were set as the NC group, with blood glucose <250 mg/dl. Another 30 10-week-old NOD mice were purchased and the blood glucose was monitored continuously for 5 days. Animals with blood glucose higher ≥250 mg/dl and were regarded as T1D model. The model mice were further divided into two groups: 1) the T1D model group and 2) the islet transplantation group (T1D model + islet). All animal experiments have been approved by the Ethics Committee of our medical school. Repeat the test at least three times.

### Islet transplantation

Islets were isolated from the above NOD mice by collagenase digestion as previously reported [30]. Generally, 2 mL collagenase type V solution was injected into the pancreatic duct and the pancreas were digested at 37° C for 15 min. The islet was isolated by Ficoll gradient (GE Health Care, USA) and cultured d in RPMI 1640 medium (Gibco, USA) supplemented with 10% FBS (Gibco, USA) and 1% penicillin and streptomycin (Gibco, USA). The islet cell equivalent was 150 μmol/L. 24 h before islet transplantation, islet was transplanted into the above NOD mice via the portal vein as previously reported [31]. Each mouse received about 0.1 mL islet. The animal experiment was repeated at least three times. In each repeat, there were 12 female 4-week-old NOD mice in the NC group, 12 10-week-old NOD mice in the T1D model group and 12 10-week-old NOD mice in the T1D model + islet group. The blood glucose levels of the T1D model group and T1D model + islet group were higher than 250 mg/dl and the blood glucose levels of the NC group was <250 mg/dl.

### The abdominal aorta blood and islet grafts from the abdominal aorta

After 12 days of islet transplantation, the mice were anesthetized and the abdominal aorta the abdominal aorta blood and pancreatic tissues were collected in each group and maintained in the 4° C refrigerator for the following bioassays.

### ELISA assay

The levels of IL-1Rα, IL-1α, IL-1β, CXCL2, MCP1, TNF-α and IL-10 in the abdominal aorta blood were detected by ELISA Kits (ThermoFisher Scientific, USA) according to the guidelines. Simply, all reagents and samples were prepared to RT (room temperature) before use. The abdominal aorta blood samples were added into the 96-well plate and were incubated at RT for 2 h. The samples were then discarded and washed for three times with washing buffer. Antibodies including IL-1Rα, IL-1α, IL-1β, CXCL2, MCP1, TNF-α and IL-10 (Cell Signaling Technology, USA) were then added to each well respectively, following with incubation at RT for 1 h. The antibody solutions were then discarded and washed for three times. Streptavidin solution was then added to each well and the samples were further incubated at RT for 1 h. Then, TMB One -Step Substrate Reagent (ThermoFisher Scientific, USA) was added to each well, with incubation at RT for 1 h. After discarding and washing, Stop Solution (ThermoFisher Scientific, USA) was added and absorbance was measured at 450 nm as soon as possible. This experiment was performed in triplicates and the average was applied to plot the columns.

### Flow cytometry

Each group of abdominal aorta blood samples containing macrophages were washed and treated with BD Pharm Lyse (BD Biosciences, San Jose, CA, USA). Inactive cells were excluded by trypan blue. The live cells were incubated with MHC-II-APC (BD Biosciences, San Jose, CA, USA) and CD-206-PE (BD Biosciences, San Jose, CA, USA). The fluorescent stained cells were analyzed by a BD LSR II flow cytometer using BD FACSDiva software (BD Biosciences, San Jose, CA, USA). And the fluorescent compensation and data analysis were performed by FlowJo 7.5 software (TreeStar, Ashland, OR). MHCII^+^CD206^-^ cells were indicated the M1 macrophages while MHCII^+^CD206^+^ cells were M2 macrophages. This assay was repeated at least three times.

### HE staining

The cryosections of islet grafts were rinsed with deionized water and stained with hematoxylin (Sigma, USA) at RT for 1 min, after washing three times with tap water, these sections were stained with eosin for 30 seconds. Then, theses sections were dehydrated with 95% ethanol and 100% ethanol (Sigma, USA) for 3 min consecutively. Finally, these sections were sealed with coverslip slides and permount after washing by tap water. The HE images were obtained under microscopes and analyzed by Image J. This assay was repeated at least three times.

### IHC (Immunohistochemistry)

The rinsed section of islet grafts was immersed into 35 H_2_O_2_ (Sigma, USA) for 10 min at RT to block endogenous peroxidase activity. After washing the sections three times with PBS, these sections were sealed with blocking buffer (ThermoFisher Scientific, USA), and then the primary antibodies of IGF1R, FGFR2 or VEGFA (Cell Signaling Technology, USA) were applied to these sections and incubated in a humidified chamber at RT for two h. These sections were incubated with biotinylated secondary antibodies (Cell Signaling Technology, USA) at RT for 1 h after washing as usual. Then, these sections were incubated with Sav-HRP conjugates (Cell Signaling Technology, USA) at RT for 30 min in the darkness. Next, DAB substrate solution (Cell Signaling Technology, USA) was added to these sections to reveal the color of IHC staining. Then, these sections were counterstained with hematoxylin (Cell Signaling Technology, USA) for 1 min. After rinsing with the running tap water, these sections were dehydrated with 95% ethanol, 100% ethanol as previously mentioned. Finally, the images of these section were obtained under microscopy and analyzed with Image J software. This assay was repeated at least three times.

### TUNEL staining

TUNEL assay was conducted for cell apoptosis in islet grafts or pancreatic islets. Briefly, tissue samples were dewaxed, washed by PBS, added protease K and incubated for 30 min at 37° C. Samples were then stained with an *In Situ* Cell Apoptosis Detection Kit (Roche Applied Science) according to the manufacturer’s instructions. The number of TUNEL positive cells was calculated as percent of total number of cells. An inverted microscope (Olympus) was used to observe the results.

### Western blot

The total protein was extracted from islet grafts and quantified by BCA assay (ThermoFisher Scientific, USA). 20 μg total protein was loaded on 10% SDS-PAGE (ThermoFisher Scientific, USA). Next, these proteins were transferred to PVDF membrane (ThermoFisher Scientific, USA). After blocking by 5% defat milk prepared by 1×TBST (Sigma, USA), the membrane was incubated with the specific primary antibodies of IGF1R, GRB2, EGFR, PTPN1, JAK2, STAT3, Caspase-1, Bcl2 or actin (Cell Signaling Technology, USA) respectively at 4° C overnight. When the membrane was washed three times with 1×TBST, it was treated with HRP-conjugated secondary antibodies (Cell Signaling Technology, USA) at RT for 1 h. Finally, the protein bands were observed after being treated with ECL solution (ThermoFisher Scientific, USA). This assay was repeated at least three times.

### Statistical analysis

The data in this study was presented as mean ± standard deviation (SD). And statistical analysis was performed by SPSS 22.0 software (SPSS Inc., Chicago, IL, USA). *T*-test was used to analyze the difference between two groups and one-way ANOVA was utilized for the difference between multiple groups. P<0.05 was considered to be statistically significant.
